# Oxidative Stress and Aberrant Programmed Cell Death Are Associated With Pollen Abortion in Isonuclear Alloplasmic Male-Sterile Wheat

**DOI:** 10.3389/fpls.2018.00595

**Published:** 2018-05-04

**Authors:** Zihan Liu, Xiaoyi Shi, Sha Li, Lingli Zhang, Xiyue Song

**Affiliations:** College of Agronomy, Northwest A&F University, Yangling, China

**Keywords:** cytoplasmic male sterility, gene expression, ROS metabolism, tapetal programmed cell death, wheat (*Triticum aestivum* L.)

## Abstract

Cytoplasmic male sterility is crucial for the utilization of hybrid heterosis and it possibly occurs in parallel with tapetal programmed cell death (PCD) and oxidative metabolism responses. However, little is known about the mechanisms that underlie pollen abortion in wheat. Therefore, we obtained two isonuclear alloplasmic male sterile lines (IAMSLs) with *Aegilops kotschyi* and *Ae. juvenalis* cytoplasm. Compared with the maintainer line, cytochemical analyses of the anthers demonstrated that the IAMSLs exhibited anomalous tapetal PCD and organelles, with premature PCD in K87B1-706A and delayed PCD in Ju87B1-706A. We also found that the dynamic trends in reactive oxygen species (ROS) were consistent in these two IAMSLs during anther development and they were potentially associated with the initiation of tapetal PCD. In addition, the activities of ROS-scavenging enzymes increased rapidly, whereas non-enzymatic antioxidants were downregulated together with excess ROS production in IAMSLs. Real-time PCR analysis showed that the expression levels of superoxide dismutase, catalase, and ascorbate peroxidase genes, which encode important antioxidant enzymes, were significantly upregulated during early pollen development. Thus, we inferred that excessive ROS and the abnormal transcript levels of antioxidant enzyme genes disrupted the balance of the antioxidant system and the presence of excess ROS may have been related to aberrant tapetal PCD progression, thereby affecting the development of microspores and ultimately causing male sterility. These relationships between the mechanism of PCD and ROS metabolism provide new insights into the mechanisms responsible for abortive pollen in wheat.

## Introduction

Hybrid vigor, or heterosis is a phenomenon where the offspring derived from crosses between two inbred plants outperform their parents. Most of the production of major crops derives from hybrid varieties, which can improve the yield by 15–50% compared with inbred varieties. Thus, the use of hybrid breeding has brought tremendous economic benefits to global crop production (Tester and Langridge, 2010). Wheat is the most widely grown crop throughout the world and one of the top three global crops, but the rate of yield increases in wheat is lower than that in rice and maize (Ray et al., 2013). Therefore, hybrid wheat is needed to obtain superior traits with better quality, higher yields, accelerated rates of growth and development, improved biomass, and greater resistance to biotic and abiotic stresses (Singh et al., 2015). Cytoplasmic male sterility (CMS) is a form of male sterility caused by an interaction between the mitochondria and the nucleus, which leads to abnormal anther development and pollen abortion. CMS has been utilized frequently in hybrid wheat breeding throughout the world (Jenny and Kristina, 2011). Studies of heterogeneous male sterile wheat lines began in 1951 when a CMS line was first generated with *Aegilops caudata*. Subsequently, more than 130 nuclear and cytoplasmic hybrids were obtained, including T, K, V, D, Mu, A, and P-type sources. Moreover, the genus *Aegilops* has been identified as the most successful genus in the Triticeae for distant hybridization with wheat (Gong et al., 2014). In previous studies, we demonstrated that the isonuclear alloplasmic male sterile lines (IAMSLs) comprising K87B1-706A with *Aegilops kotschyi* (K-type) cytoplasm and Ju87B1-706A with *Ae. juvenalis* cytoplasm (D^2^-type) have many advantages for the production and utilization of hybrid wheat, where they are easy to restore, as well as increasing the growth potential, improving the wheat quality, and enhancing powdery mildew resistance (Zeng et al., 2013; Yao et al., 2015). Furthermore, they can lead to complete male sterility. Thus, these two types of IAMSLs are of great value in the breeding and production of hybrid wheat, and they can also provide ideal materials for studying reproductive growth, cytoplasmic inheritance, and pollen development. The IAMSLs have few detrimental effects on agronomic traits (Xu et al., 2001) but the abortive mechanism is still unclear.

Programmed cell death (PCD) is a genetically controlled process that occurs in both animal and plant cells, and it has attracted much attention recently (Wang et al., 2009; Dutcher et al., 2016; Preyat and Leo, 2016). PCD plays an essential role in various processes such as responses to stresses, including leaf senescence (Wang et al., 2016), the removal of aleurone cells (Zheng et al., 2016), root cap cells (Xuan et al., 2016), and xylogenesis (Iakimova and Woltering, 2017). Previous studies have suggested that the tapetal degradation process in the anthers matches with the basic characteristics of PCD, and PCD is also of special importance for tapetal development in anthers (Solís et al., 2014; Wang et al., 2015; Meng et al., 2016). During anther development, the tapetum plays a key secretory role in timely PCD by contributing to the nutrients, enzymes, and sporopollenin precursors required for pollen wall synthesis and pollen coat deposition. However, premature or delayed PCD by tapetal cells will disorganize the supply of nutrients to the microspores, thereby resulting in pollen abortion (Li et al., 2011; Song et al., 2015). Recently, studies of mitochondria-mediated tapetal PCD have made some progress (Xie et al., 2014). It is generally considered that the PCD process is associated with reactive oxygen species (ROS) and growing evidence demonstrates that ROS can act as a signal or participate in PCD. Thus, attention has focused on the effects of biotic and abiotic stresses on PCD in microorganisms, insects, animals, and plant leaves (Farrugia and Balzan, 2012; Mishra and Singh, 2013; Murik et al., 2014; Ruberti et al., 2014). However, few studies have considered the relationship between ROS and natural tapetal PCD in the anthers of CMS wheat, and it is unknown whether the mechanism exists in the two most important types of IAMSL wheat.

In aerobic organisms, mitochondria provide energy for all of the activities that occur in cells via respiration. In addition, mitochondria can generate ROS including the superoxide anion radical (O_2_^-^) and hydrogen peroxide (H_2_O_2_). H_2_O_2_ possesses a signal function as a secondary messenger where it participates in the regulation of development and growth as well as apoptosis of the tapetum. However, after the accumulation of ROS increases to an excessive level, the organism will suffer oxidative stress, which may cause damage to proteins and nucleic acids, lipid peroxidation, and even necrocytosis (Shadel and Horvath, 2015). Malondialdehyde (MDA) is a marker of oxidative lipid injury because its concentration varies in response to biotic and abiotic stresses (Li et al., 2012). The mitochondrion is also a major site for maintaining the redox homeostasis between ROS generation and scavenging (Dutilleul et al., 2003). Thus, this intracellular balance requires the efficient coordination of complex antioxidant systems comprising enzymatic antioxidants such as superoxide dismutase (SOD), peroxidase (POD), catalase (CAT), ascorbate peroxidase (APX), and glutathione peroxidase (GPX), as well as non-enzymatic scavengers such as ascorbic acid (AsA) and glutathione (GSH) (Wang et al., 2016). Normally, SOD can promote the conversion of O_2_^-^ to H_2_O_2_ and O_2_, POD can disintegrate H_2_O_2_ to O_2_, and CAT, APX, and GPX can dismutate H_2_O_2_ into H_2_O to create a relatively balanced redox environment (Arora et al., 2002). However, it has been documented that the excessive accumulation of ROS leads to oxidative stress, and thus oxidative signaling by ROS affects homeostasis in the redox and antioxidant systems, thereby causing male sterility (Apel and Hirt, 2004; Wan et al., 2007). Hence, ROS and the antioxidant system play important roles in the maintenance of cellular redox homeostasis in CMS.

Many studies have reported that CMS is related to abnormal mitochondria (Shen et al., 2011; Zhang et al., 2012), where abnormal mitochondria often lead to abnormal respiratory metabolism in CMS (Peng et al., 2009; Jacoby et al., 2010). Numerous studies have also shown that ROS metabolism changes when plant tissues are subjected to stress and that CMS lines accumulate excessive ROS (Li et al., 2004; Wan et al., 2007). Nevertheless, the metabolic characteristics and molecular mechanisms of ROS generation and scavenging as well as their relationships with abnormal PCD are still unclear. Therefore, it is important to study the relationships between ROS, the activities of antioxidant and non-enzymatic enzymes, the expression levels of related genes, and abnormal PCD to identify the mechanism responsible for CMS, thereby obtaining insights into the pollen abortion mechanism in wheat.

The abortive mechanism that determines male sterility in wheat is extremely complex and different types of CMS can only be compared objectively when the factors related to the nucleus are excluded, but few studies have attempted this type of analysis. In the present study, we investigated the characteristics of two IAMSLs with identical nuclear genomes but different cytoplasmic sources, thereby eliminating the influence of the nucleus on our cytological, physiological, and molecular analyses in order to explore the mechanism of male abortion, as well as providing a theoretical basis for the application of sterile wheat lines. We compared morphological and tapetal changes during different developmental stages based on paraffin sections, semithin sections, transmission electron microscopy (TEM) observations, terminal deoxynucleotidyl transferase-mediated dUTP nick-end labeling (TUNEL) assays, and DNA laddering analysis, where we identified the period of abortion based on iodine-potassium iodide (I_2_–KI) staining, scanning electron microscopy (SEM) observations, and 4′,6-diamidino-2-phenylindole (DAPI) staining. We also elucidated the abortive mechanism in the IAMSLs by determining physiological indices and based on quantitative real-time PCR (qRT-PCR). The results of this study provide new insights into the relationships between the mechanism of PCD and ROS metabolism in IAMSLs used for hybrid breeding.

## Materials and Methods

### Plant Materials

This study used two types of IAMSLs, i.e., K87B1-706A with *Aegilops kotschyi* cytoplasm and Ju87B1-706A with *Ae. juvenalis* cytoplasm, as well as their maintainer line, 706B, which had the same nuclear background. These sterile lines were developed from stable sterile lines by backcrossing with 706B over 20 times in Yangling (108°E, 34°15′N), China. To check the stability of sterility in the IAMSLs, the IAMSLs (K87B1-706A and Ju87B1-706A) were planted in wheat fields during October 2014 at the experimental stations of Northwest A&F University, Yangling, China. In April 2015, each of the IAMSLs was checked by bagging. The results showed that the self-setting rates of these lines were all zero, and thus the male sterile lines were stable, thereby confirming our previous studies (Yao et al., 2015). The dates of the major growth stages and young panicle differentiation stages were recorded for the IAMSLs and their maintainer line 706B.

### Phenotypic Characterization of IAMSLs and Their Maintainer Line

The microspore development stages were analyzed by acetic acid magenta staining of the nuclei and microscopy, as described previously (Ba et al., 2013). The overall anther development period was divided into five stages according to the progress of microsporogenesis: tetrad stage (Tds), early uninucleate stage (Ens), late uninucleate stage (Lns), binucleate stage (Bns), and trinucleate stage (Tns). Spikelets and anthers from the five microspore developmental stages were observed and photographed using a Motic K400 stereomicroscope.

To assess the viability of mature pollen, the pollen grains from anthers in the trinucleate stage were stained using I_2_–KI (Chang et al., 2009). Anthers and microspores in the trinucleate stage were collected, fixed in 2.5% glutaraldehyde, dehydrated, dried (Zhang et al., 2007), and observed by SEM (JSM-6360LV; JEOL). The I_2_–KI staining results and the numbers of Ubisch bodies were counted using IPP 6.0 software.

### Observations of Microspores and Anther Tapetum

The morphology of the pollen grains was observed in various microspore development stages by staining the nuclei with DAPI before microscopy (Zhang et al., 2013). In order to observe the tapetum development process, anthers at different developmental stages were fixed in formalin–acetic acid–ethanol and embedded in paraffin wax. Transverse sections measuring 8 μm were placed onto poly-L-lysine-coated slides (Sigma-Aldrich) and stained with toluidine blue (Wang et al., 2015). To obtain further detailed observations of the tapetal cells, anthers at different developmental stages were prefixed in 2.5% glutaraldehyde and embedded in glue, as described by Zhang et al. (2014). Semithin sections of 1 μm were cut using a UC6 ultramicrotome (Leica) and stained with 1% toluidine blue O (Sigma-Aldrich). All of the samples were photographed using a DS-U2 high resolution camera mounted on a Nikon ECLIPSE E600 microscope and processed with NIS-Elements software. The cellSens Entry software (Olympus, Japan) was utilized to calculate the area of the tapetum cells in the section. Ultrathin sections (60 nm) were obtained with a UC6 ultramicrotome (Leica), before staining with 2% (w/v) uranyl acetate and 2.6% (w/v) lead citrate aqueous solution. Finally, we used an H-7650 transmission electron microscope (Hitachi) and an 832 charge-coupled device camera (Gatan) to observe and capture all of the TEM images (Zhang et al., 2014).

### TUNEL Assay

Transverse paraffin sections were dehydrated twice in 100% xylol and washed in a graded ethanol series (100% twice, 95%, 90%, 80%, 70%, and 0% twice), before incubating with 20 μ mL^-1^ Proteinase K (Roche) for 15 min, and then washing in phosphate-buffered saline (PBS; pH = 7.4) for 15 min. *In situ* nick-end labeling of the nuclear DNA fragments was implemented by placing the mixed solution (TdT: dUTP = 1:9) in a humid chamber for 60 min at 37°C in the dark. All of the sections were placed in PBS (pH = 7.4) and washed three times, each for 5 min. All of the reactions were counterstained with DAPI and incubated for 10 min in the dark. Samples were analyzed using a fluorescence confocal scanner microscope (A1R; Nikon, Tokyo, Japan). The emission/excitation wavelengths of the TUNEL signals and DAPI signals were 450–500 nm/515–565 nm and 358 nm/461 nm, respectively (Wang et al., 2015).

### DNA Laddering Analysis

Total DNA was isolated from the anthers in different developmental stages using the cetyl trimethylammonium bromide extraction procedure (Hu et al., 2016). Next, the total DNA was redissolved in Tris-EDTA buffer solution (10 mmol L^-1^ Tris-HCl (pH 8.0), 5 mmol L^-1^ EDTA) and incubated at 37°C for 60 min in the presence of RNase A (100 g mL^-1^). Subsequently, 10 μg of DNA was separated by electrophoresis on a 1.8% (w/v) TBE-agarose gel. The gel was then stained with ethidium bromide to visualize the DNA ladder (Wang et al., 2016).

All of these observations (anthers, I_2_–KI, SEM, paraffin sections, semithin sections, TEM, TUNEL, and DNA ladders) were obtained based on three replicates for each sample.

### Quantification of Physiological Indices

Anthers (0.5 g) in different developmental stages were collected to determine the physiological indexes. The anthers were ground with 5 mL of pre-chilled extraction buffer [0.1 M Tris-HCl (pH 8.0); 0.5 mM EDTA; 0.1% (v/v) Triton X-100; 1% PVP]. The mixture was centrifuged at 12,000 × *g* and 4°C for 10 min. The supernatant was collected and centrifuged again at 12,000 × *g* and 4°C for 10 min. Finally, we used the supernatant to analyze the O_2_^-^, H_2_O_2_, and MDA contents, as well as the antioxidant enzymes (SOD, POD, CAT, and GPX) according to Ba et al. (2013). The APX activities were assayed using the method described by Miyake et al. (2006). The AsA contents were determined according to Wang et al. (2009). The GSH contents were calculated by subtracting the glutathione disulfide contents from the total GSH contents (Jiang and Zhang, 2001). Three biological replicates for all developmental stages were conducted for each material.

### Diphenyleneiodonium Chloride (DPI) Treatment

DPI stock solution was prepared and diluted with 0.02% (w/v) Triton X-100 as a 10 μM working solution. The DPI was applied during the meiosis period in the IAMSL plants, where it was sprinkled on the surfaces of the anthers once each day until the trinucleate stage. The anthers were then collected in the tetrad stage, early uninucleate stage, late uninucleate stage, binucleate stage, and trinucleate stage. The total DNA was isolated from the DPI-treated anthers in different developmental stages and subjected to DNA laddering analysis (Yu et al., 2017).

### Quantitative Real-Time-PCR (qRT-PCR) Analysis of Antioxidant Enzyme Genes

Total RNA was extracted using the Trizol procedure (Tiangen, China). cDNA was synthesized using HiScript^TM^ Q Select RT SuperMix for qRT-PCR (Vazyme, China). The qRT-PCR reaction products were analyzed with a QuantStudio^TM^ Real-Time PCR System (Applied Biosystems, United States) using AceQ^®^ qRT-PCR SYBR^®^ Green Master Mix (Vazyme, China), according to the manufacturer’s protocols. qRT-PCR reactions were performed with the specific primers shown in Supplementary Table S1. The *TaActin* gene was used as an internal control. The reaction was pre-denatured at 95°C for 2 min, followed by 40 cycles of heating at 95°C for 15 s and annealing at 60°C for 1 min. The relative expression levels of genes were calculated using the 2^-ΔΔCt^ method. All of the experiments were performed in triplicate (Udvardi et al., 2008; Xie et al., 2014).

### Statistical Analysis

The experiments (I_2_–KI staining, counts of Ubisch bodies, area of the tapetum cells, ROS analyses, and enzymatic and non-enzymatic analyses) were performed in dependent series with three replicates in order to reduce errors. Statistical analyses were conducted between different stages of anther development for each experiment using one-way analysis of variance. Tests of significant differences and correlation analyses were performed using SPSS statistical software and Excel Office. The results were expressed as the mean ± standard deviation.

## Results

### Abortive Morphological Features in Wheat IAMSLs

**FIGURE 1:**
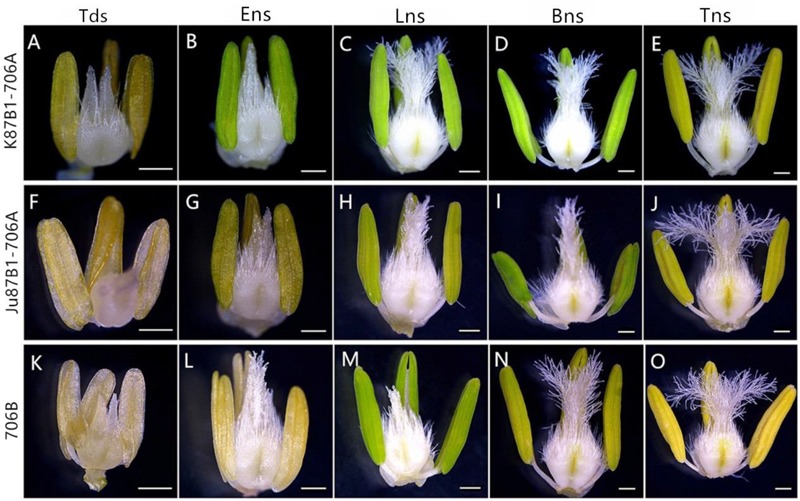
Comparisons of the stamens and pistils in K87B1-706A **(A–E)**, Ju87B1-706A **(F–J)** and 706B **(K–O)**. Tds, tetrad stage **(A,F,K)**; Ens, early uninucleate stage **(B,G,L)**; Lns, late uninucleate stage **(C,H,M)**; Bns, binucleate stage **(D,I,N)**; and Tns, trinucleate stage **(E,J,O)**. Scale bars = 0.5 mm **(A–O)**.

**FIGURE 2:**
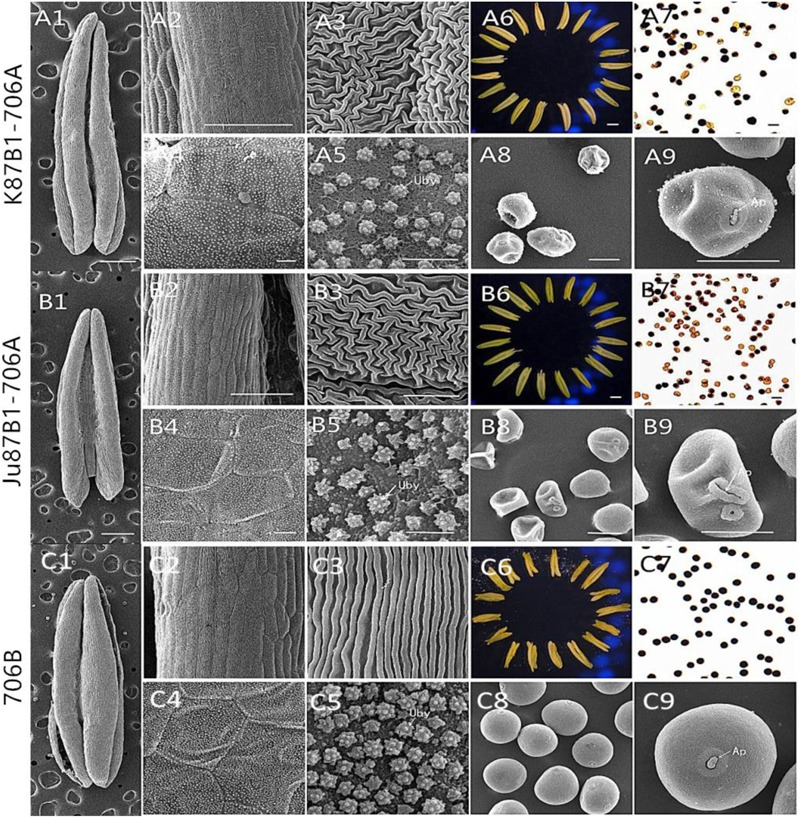
Comparison of scanning electron micrograph observations, I_2_–KI staining, and anther morphology in K87B1-706A **(A1–A9)**, Ju87B1-706A **(B1–B9)**, and 706B **(C1–C9)** during the trinucleate stage. Anthers **(A1,A6,B1,B6,C1,C6)**. Outer epidermal cells **(A2,A3,B2,B3,C2,C3)**. Inner epidermal cells **(A4,A5,B4,B5,C4,C5)**. Uby, Ubisch bodies. Microspores by I_2_-KI staining **(A7,B7,C7)**. Microspores **(A8,A9,B8,B9,C8,C9)**. Ap indicates the germination aperture. Scale bars = 1 mm **(A1,A6,B1,B6,C1,C6)**; 100 μm **(A2,B2,C2)**; 50 μm **(A7–A9,B7–B9,C7–C9)**; and 10 μm **(A3–A5,B3–B5,C3–C5)**.

According to preliminary acetic acid magenta staining observations of microspore development, we assigned wheat anther development to five stages. The anthers from the two types of IAMSLs all appeared normal in the tetrad stage and the early uninucleate stage (**Figures 1A,B,F,G,K,L**). However, from the late uninucleate stage until the trinucleate stage, the anthers of the IAMSLs were all smaller and their color was lighter than those of the maintainer line (**Figures 1C–E,H–J,M–O**). Importantly, in the trinucleate stage, the anthers from fertile plants were dehiscent with shedding of the mature pollen grains (**Figures 2C1, C6**). However, the anthers of the IAMSLs were not cracked in the trinucleate stage, and the anthers released little or no pollen when mature (**Figures 2A1,A6,B1,B6**).

According to I_2_–KI staining, pollen abortion was divided into three types in the trinucleate stage: typical abortion, round abortion, and stainable abortion. The main features of typical abortion were a lack of dyeing and an irregular shape. In round abortion, the pollen was rounded without inclusions. In stainable abortion, the pollen was round and the staining was not sufficient. By contrast, fertile pollen was rounded with full and deep dyeing. The results demonstrated that unlike the maintainer line, the pollen abortion types of the IAMSLs all comprised typical and stainable abortion, and the plants were 100% pollen sterile (**Figures 2A7,B7,C7**), but the percentages of typical and stainable abortive pollen grains were different. The typical abortive percentages in K87B1-706A and Ju87B1-706A were 20.6 and 52.4%, respectively, and the stainable abortive percentages were 79.4 and 47.6% (Supplementary Figure S1).

To further understand the abnormalities in the anthers of the IAMSL plants at the trinucleate stage, SEM was used to observe the surfaces of the inner and outer epidermis in the anthers. The outer epidermal cells in the anthers were more shrunken than those in the fertile plant cells, and they had irregular shapes (**Figures 2A2,A3,B2,B3,C2,C3**). In addition, compared with the maintainer line 706B, the inner epidermal cells of the IAMSLs accumulated lesser amounts and more unconsolidated Ubisch bodies, which may have an adhesive role (**Figures 2A4,A5,B4,B5,C4,C5** and Supplementary Figure S2). Based on observations of the epidermis of the microspores and the germinal aperture, we found that the epidermis was more rounded and plump with a gymnotremoid germinal aperture in the fertile microspores, whereas the epidermis was crenate and extremely scabrous with a malformed germinal aperture in the IAMSL microspores (**Figures 2A8,A9,B8,B9,C8,C9**).

These results indicate that the IAMSLs were 100% pollen sterile and the anthers were severely detrimentally influenced during the development of the microspores in K87B1-706A and Ju87B1-706A.

### Cytological Characteristics of Pollen Grains

**FIGURE 3:**
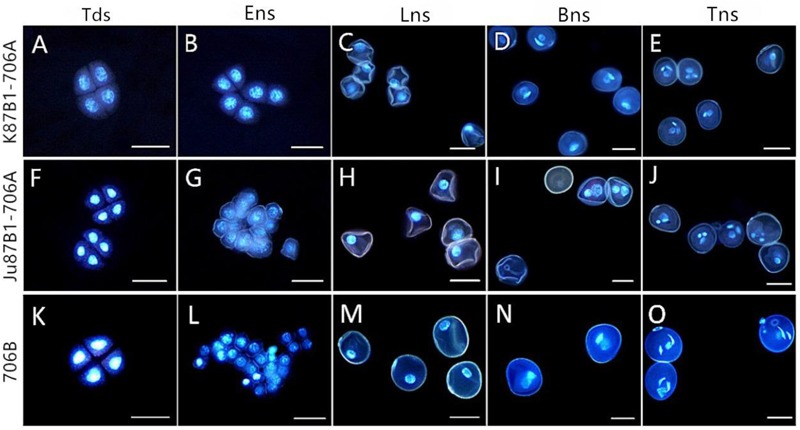
DAPI staining of microspores in K87B1-706A **(A–E)**, Ju87B1-706A **(F–J)**, and 706B **(K–O)**. Tds, tetrad stage **(A,F,K)**; Ens, early uninucleate stage **(B,G,L)**; Lns, late uninucleate stage **(C,H,M)**; Bns, binucleate stage **(D,I,N)**; and Tns, trinucleate stage **(E,J,O)**. Scale bars = 50 μm **(A–O)**.

To investigate the cytological characteristics of the IAMSLs, we analyzed the development of microspores using DAPI staining in a range of developmental stages (**Figure 3**). In the tetrad stage, meiosis progressed normally in 706B and the IAMSLs, where flabellate tetrads formed (**Figures 3A,F,K**). In the early uninucleate stage, the microspores were released from the tetrads in 706B and the IAMSLs, and they exhibited normal development where the nuclei were all located at the center of the cell with no differences (**Figures 3B,G,L**). In the late uninucleate stage, the microspores began to form huge vacuoles in 706B and the IAMSLs, and the nuclei were all pushed to the opposite side of the germination aperture, but the shape of the microspores was severely irregular in the IAMSLs compared with those in 706B (**Figures 3C,H,M**). During the binucleate stage, the microspores continued to enlarge, where they rounded and formed compact vegetative and sperm nuclei in 706B. In contrast to 706B, the vegetative and sperm nuclei were slightly bigger in the IAMSLs, and some of the vegetative nuclei were not clear (**Figures 3D,I,N**). In the trinucleate stage, the sperm nuclei were round in the IAMSLs instead of being fusiform in shape and some of the spores were empty (**Figures 3E,J,O**).

Based on these results, we suggest that the pollen abortion period was almost identical in the IAMSLs, where it always commenced at the late uninucleate stage.

### Tapetum Observations During Microspore Development

**FIGURE 4:**
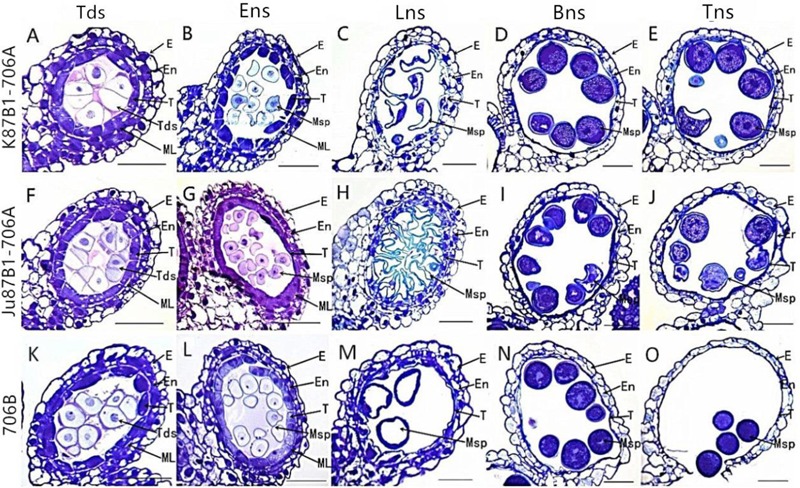
Comparisons of the anther tapetum in K87B1-706A **(A–E)**, Ju87B1-706A **(F–J)**, and 706B **(K–O)** during different developmental stages. Tds, tetrad stage **(A,F,K)**; Ens, early uninucleate stage **(B,G,L)**; Lns, late uninucleate stage **(C,H,M)**; Bns, binucleate stage **(D,I,N)**; and Tns, trinucleate stage **(E,J,O)**. E, epidermis; En, endothecium; ML, middle layer; T, tapetum; Tds, tetrads; Msp, microspores. Scale bars = 50 μm **(A–O)**.

To further investigate the causes of pollen abortion and aberrant variations in the tapetum in the IAMSLs, we performed detailed examinations of variations in the tapetum based on paraffin sections, semithin sections, and using cellSens Entry software. Compared with 706B, light microscopy observations of K87B1-706A in the tetrad stage showed that the tapetal cells were smaller than those in 706B and the cytoplasm did not stain as deeply with toluidine blue as the 706B cells (**Figures 4A,K** and Supplementary Figures S3A,K, S4). During the early uninucleate stage, the tapetal cells were partially detached from the middle layer and they began to degrade earlier than those in 706B (**Figures 4B,L** and Supplementary Figures S3B,L, S4). The tapetal cells continued to degrade until the later uninucleate stage and their outline was almost imperceptible (**Figures 4C,M** and Supplementary Figures S3C,M, S4). In the binucleate stage, the tapetum had degenerated completely after its function was complete (**Figures 4D,N** and Supplementary Figures S3D,N). In the trinucleate stage, the anther wall was fractured and this finally led to the complete disintegration of the tapetum, while the invasion of the cell mass could also cause the abortion of microspores (**Figures 4E,O** and Supplementary Figures S3E,O). Based on these findings, we suggest that the advanced dissociation of the tapetum may be related to pollen abortion in K87B1-706A. There were no obvious differences in the cellular morphology of the anthers in 706B and Ju87B1-706A during the tetrad and early uninucleate stages (**Figures 4A,B,F,G** and Supplementary Figures S3A,B,F,G, S4). However, in contrast to K87B1-706A, the areas of the tapetal cells were larger in Ju87B1-706A than those in 706B during the later uninucleate stage, where they occupied the majority of the locule and they were arranged neatly instead of degenerating to allow microspore formation (**Figures 4H,M** and Supplementary Figures S3H,M, S4). Thus, most of the microspores failed to develop due to a lack of nutrition because of the failed dissociation of the tapetum. When the mature pollen grains formed, the tapetum degenerated completely in the same manner as K87B1-706A in the binucleate and trinucleate stages (**Figures 4I,J,N,O** and Supplementary Figures S3I,J,N,O). Therefore, we suggest that the delayed initiation of tapetum dysfunction possibly resulted in the abortion of Ju87B1-706A.

**FIGURE 5:**
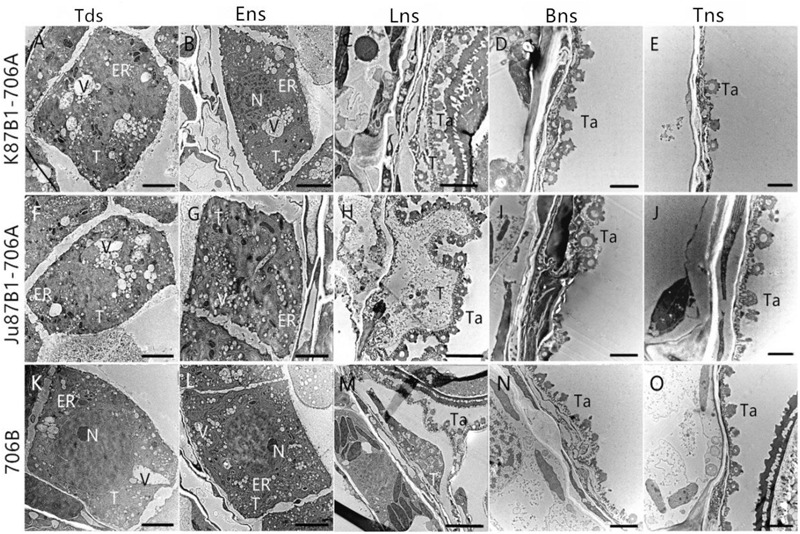
Transmission electron micrographs of the anthers in K87B1-706A **(A–E)**, Ju87B1-706A **(F–J)**, and 706B **(K–O)** during different developmental stages. Tds, tetrad stage **(A,F,K)**; Ens, early uninucleate stage **(B,G,L)**; Lns, late uninucleate stage **(C,H,M)**; Bns, binucleate stage **(D,I,N)**; and Tns, trinucleate stage **(E,J,O)**. ER, endoplasmic reticulum; N, nucleus; T, tapetum; Ta, tapetosome; V, vacuole. Scale bars = 2 μm **(A–O)**.

To confirm the results based on the semi-thin sections and to further understand the changes in the organelles in the tapetal cells, TEM was performed for the tapetum in the IAMSLs during all of the developmental stages. Consistent with the semi-thin section results, the tapetal cells were still fairly intact in the tetrad and early uninucleate stages in IAMSLs, although the nuclear chromatin was more blurred, the nuclear membranes had disappeared to a greater extent, with more vacuoles, and the endoplasmic reticulum was expanded more than that in 706B (**Figures 5A,B,F,G,K,L**). From the later uninucleate stage to the trinucleate stage, the morphology of the tapetum cells was identical to that in the semi-thin sections, but the tapetosome was more scattered and the organelles were more indistinct compared with 706B (**Figures 5C–E,H–J,M–O**). Our observations demonstrate that abortion may be caused by anomalous changes in the organelles and abnormal tapetal degradation, which affect pollen development.

### Tapetal PCD Detection in Anthers

**FIGURE 6:**
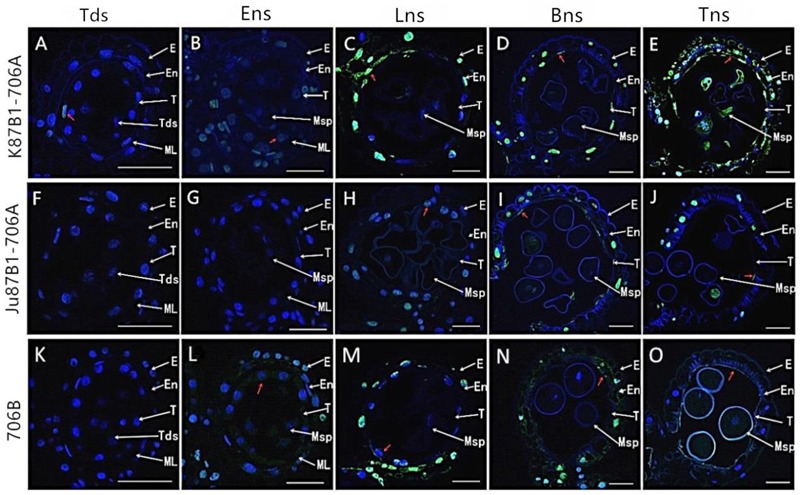
TUNEL assays to detect anther tapetum PCD in K87B1-706A **(A–E)**, Ju87B1-706A **(F–J)**, and 706B **(K–O)** during different developmental stages. Tds, tetrad stage **(A,F,K)**; Ens, early uninucleate stage **(B,G,L)**; Lns, late uninucleate stage **(C,H,M)**; Bns, binucleate stage **(D,I,N)**; and Tns, trinucleate stage **(E,J,O)**. E, epidermis; En, endothecium; ML, middle layer; T, tapetum; Tds: tetrads; Msp: microspores. The yellow fluorescence denoted by the red arrows indicates nuclei with TUNEL-positive staining. Scale bars = 50 μm **(A–O)**.

To further characterize the initiation of tapetal PCD, we studied the fragmentation of nuclear DNA using TUNEL assays in the IAMSLs and 706B in different developmental stages. In the tetrad stage, a weak TUNEL fluorescence signal was detected in K87B1-706A tapetal cells, thereby indicating that PCD of the tapetum were present in this stage (**Figures 6A,K**). In the early uninucleate stage, yellow TUNEL-positive signals were found in the 706B tapetum, thereby suggesting that the tapetal cells had started to degenerate in this stage. In addition, the K87B1-706A cells produced stronger TUNEL-positive signals, which was explained by the obvious accumulation of DNA cleavage in the early uninucleate stage (**Figures 6B,L**). Until the later uninucleate stage, TUNEL-positive signals were observed in the Ju87B1-706A tapetum (**Figures 6F–H**). Compared with Ju87B1-706A, more intense TUNEL-positive signals occurred in the degenerating tapetum in both K87B1-706A and 706B (**Figures 6C,H,M**). From the binucleate stage to the trinucleate stage, strong TUNEL-positive signals were also observed in the epidermis and the endothecium cells in the IAMSLs and 706B (**Figures 6D,E,I,J,N,O**). These observations also verified that the initiation of tapetal cell DNA fragmentation was abnormal in the IAMSLs, with advanced tapetal PCD in K87B1-706A and delayed tapetal PCD in Ju87B1-706A.

In order to quantify the TUNEL assay results, we compared the DNA damage level in the anthers of IAMSLs during different developmental stages based on DNA ladder assays. DNA isolated from 706B anthers started to migrate as a perpetuating molecular mass band of more than 140 bp in the early uninucleate stage. Unlike 706B, DNA fragmentation in K87B1-706A first appeared in the tetrad stage, whereas the DNA of Ju87B1-706A exhibited a significant degree of fragmentation and it formed ladders with bands in the later uninucleate stage (Supplementary Figure S5). The results obtained based on the DNA ladder assays indicate that the anther tapetum exhibited typical PCD characteristics, where K87B1-706A was characterized by premature PCD, whereas Ju87B1-706A exhibited deferred PCD in the tapetum. We also found that the nuclear DNA fragmentation signal was detected earlier than the cytological phenotype in all of materials.

### Relationship Between ROS and PCD

**FIGURE 7:**
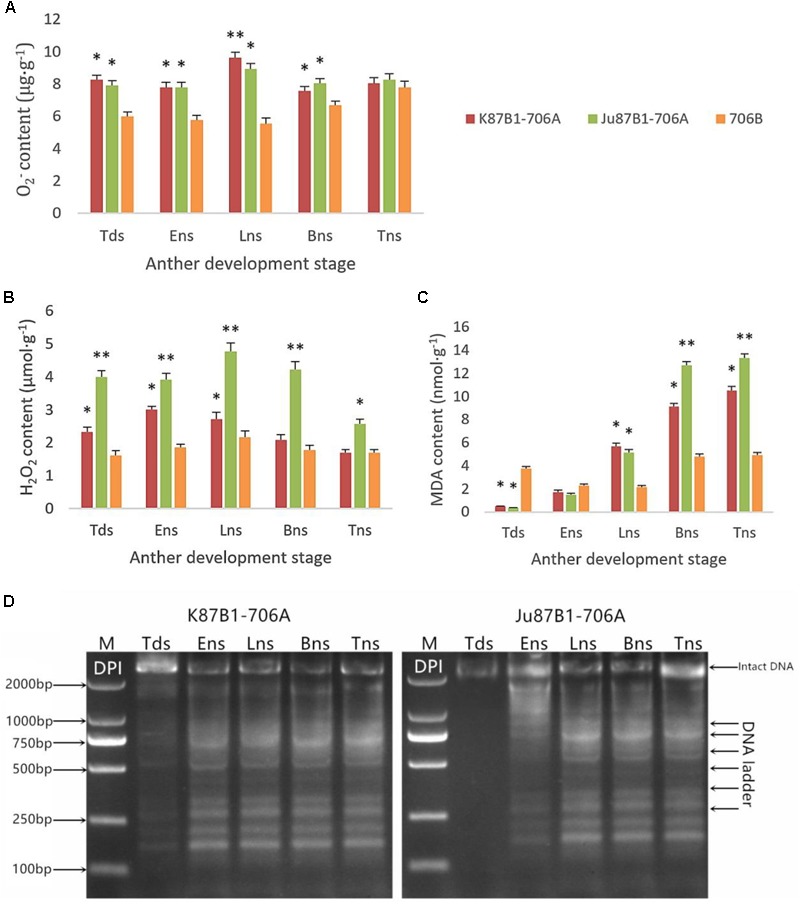
Accumulation of ROS and pharmacological interference with ROS compromised tapetal PCD in K87B1-706A, Ju87B1-706A, and 706B during various developmental stages. Tds, tetrad stage; Ens, early uninucleate stage; Lns, late uninucleate stage; Bns, binucleate stage; and Tns, trinucleate stage. O_2_^-^ contents **(A)**; H_2_O_2_ contents **(B)**; and MDA contents **(C)**. ^∗^*P* < 0.05; ^∗∗^*P* < 0.01. Detection of DNA laddering in the DPI-treated anther from K87B1-706A and Ju87B1-706A **(D)**. M, Marker D2000.

To explore the relationship between abnormal anther tapetal PCD and ROS metabolism, we determined the O_2_^-^ generation rate as well as the H_2_O_2_ and MDA contents during all of the anther developmental stages in the IAMSLs and 706B. Great increases in the O_2_^-^ and H_2_O_2_ contents occurred in the IAMSLs, with peak values in the late uninucleate stage (**Figures 7A,B**). The MDA contents of the IAMSLs increased continually and rapidly from the early uninucleate stage. In most of the stages (late uninucleate stage, binucleate stage, and trinucleate stage), the MDA contents were higher in the CMS lines than the maintainer line (**Figure 7C**). We found that excessive amounts of ROS accumulated in the IAMSLs during all the anther developmental stages, especially in the vital stage of aberrant tapetum PCD initiation, and thus we suggest that it may be related to tapetal PCD.

In order to further confirm the effect of ROS on tapetal PCD progression, DPI (ROS inhibitor) was applied to the anthers of the two IAMSLs to scavenge ROS during various developmental stages. To determine the extent of tapetal nuclear DNA fragmentation after DPI treatment, we conducted DNA ladder assays in the DPI-treated anthers. Unlike the untreated K87B1-706A anther DNA ladder assays (Supplementary Figure S5), DNA fragmentation was distinctly weaker in the treated K87B1-706A anthers than the other developmental stages and it almost disappeared. Moreover, compared with untreated Ju87B1-706A, DNA fragmentation was apparent in the early uninucleate stage in the Ju87B1-706A anthers treated with DPI (**Figure 7D**). The results showed that the Ju87B1-706A anthers treated with DPI exhibited advanced tapetal apoptosis and they tended to be consistent with the maintainer (**Figure 7D** and Supplementary Figure S5). Overall, these findings indicate that the inhibition of ROS affected the timing and progression of tapetal PCD, and thus the overproduction of ROS in IAMSLs may be related to anomalous tapetal PCD initiation, which causes male sterility.

### Activities of Non-enzymatic Antioxidants and ROS-Scavenging Enzymes

**FIGURE 8:**
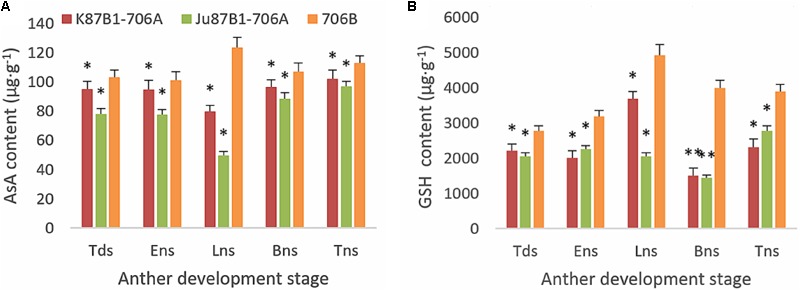
AsA and GSH contents in anthers from K87B1-706A, Ju87B1-706A, and 706B during different developmental stages. Tds, tetrad stage; Ens, early uninucleate stage; Lns, late uninucleate stage; Bns, binucleate stage; and Tns, trinucleate stage. AsA contents **(A)**; GSH contents **(B)**. ^∗^*P* < 0.05; ^∗∗^*P* < 0.01.

**FIGURE 9:**
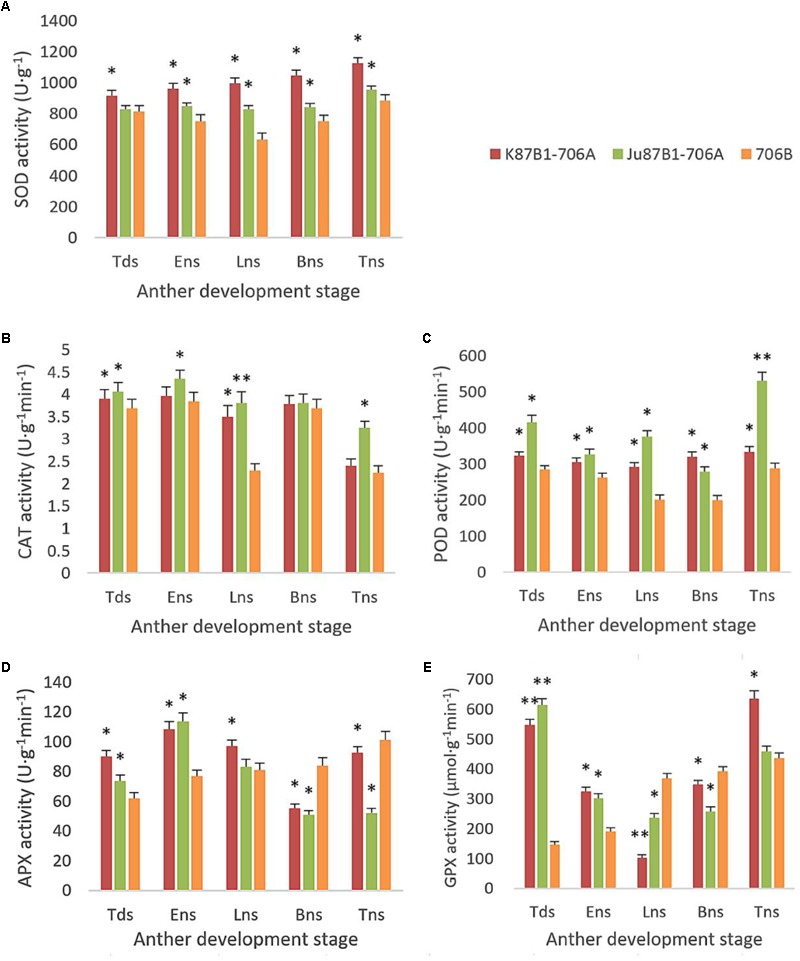
SOD, CAT, POD, APX, and GPX activities in anthers from K87B1-706A, Ju87B1-706A, and 706B during different developmental stages. Tds, tetrad stage; Ens, early uninucleate stage; Lns, late uninucleate stage; Bns, binucleate stage; and Tns, trinucleate stage. SOD activity **(A)**; CAT activity **(B)**; POD activity **(C)**; APX activity **(D)**; and GPX activity **(E)**. ^∗^*P* < 0.05; ^∗∗^*P* < 0.01.

From the tetrad stage until the late uninucleate stage, there was a sustained decline in the AsA and GSH contents of the IAMSLs, whereas they increased greatly in the later stages. By contrast, the AsA and GSH contents increased until the late uninucleate stage in the maintainer line, before a rapid decline in the later stages of anther development (**Figure 8**). The AsA and GSH contents were higher in 706B than the IAMSLs in the late uninucleate stage, where the differences between the IAMSLs and maintainer line were significant (*P* < 0.01). According to these results, we suggest that the ROS-scavenging capacity may have been weaker in the late uninucleate stage in the IAMSLs due to downregulation of the AsA and GSH levels, thereby leading to disordered activated oxygen metabolism. We also determined the activities of antioxidant enzymes, i.e., SOD, CAT, POD, APX, and GPX. The results showed that the SOD, CAT, and POD activities were always lower in the maintainer line than those in the IAMSLs throughout anther development, and there were significant differences between those in 706B and the IAMSLs in the late uninucleate stage (*P* < 0.01) (**Figures 9A–C**). The APX and GPX activities increased continually throughout anther development in the maintainer line. The APX activities were higher in 706B than those in the IAMSLs during the binucleate stage and trinucleate stage. A great decrease in the GPX activities occurred in the IAMSLs, with minimum values in the late uninucleate stage (**Figures 9D,E**). This analysis of the activities of ROS-scavenging enzymes suggests that upregulation of the activities of SOD, CAT, and POD could disrupt the balance of endogenous hormones, whereas downregulation of the activities of APX and GPX may increase the accumulation of ROS during late pollen development.

### Expression Levels of Antioxidant Enzyme Genes

**FIGURE 10:**
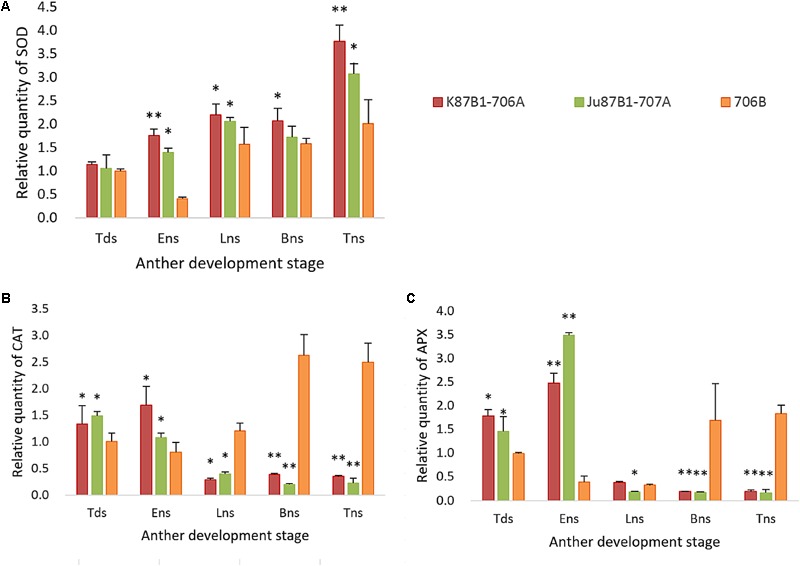
Quantitative real-time-PCR results of gene *SOD* **(A)**, *CAT* **(B)**, and *APX* **(C)** in anthers from K87B1-706A, Ju87B1-706A, and 706B during different developmental stages. Tds, tetrad stage; Ens, early uninucleate stage; Lns, late uninucleate stage; Bns, binucleate stage; and Tns, trinucleate stage. ^∗^*P* < 0.05; ^∗∗^*P* < 0.01.

The expression levels of antioxidant enzymes were evaluated in the IAMSLs and maintainer line by qRT-PCR (**Figure 10**). The SOD expression levels were higher in the IAMSLs than 706B. In the maintainer line, the *CAT* and *APX* gene expression levels increased throughout anther development. By contrast, the *CAT* gene expression levels decreased over time in Ju87B1-706A. The maximum *CAT* gene expression level occurred during the tetrad stage in Ju87B1-706A. Moreover, the *CAT* gene expression level was significantly higher in 706B than the IAMSLs in the binucleate stage and trinucleate stage (**Figure 10**). Furthermore, we employed a tendency chart and SPSS software to analyze the correlations between the enzyme activities and gene expression levels, which showed that all of the enzyme activities and the related genes expression levels were positively correlated (Supplementary Figures S6–S8). There were significant correlations between the *SOD* and *CAT* gene expression levels with the SOD and CAT enzyme activities in K87B1-706A (Supplementary Figures S6, S7). The results showed that the abnormal changes in ROS, antioxidants, and the expression of related enzymes were consistent with abnormal PCD in the anther tapetum.

## Discussion

### Cytology and Tapetal PCD in CMS

During normal pollen development, the sporogenous cells divide to generate microspore mother cells, which subsequently proceed via meiosis to form haploid microspore tetrads, before undergoing two rounds of cell division to develop into mature pollen. However, pollen abortion is an essential feature of male sterility, which can be identified in different stages of pollen development in male sterile lines (Wang et al., 2015). Some studies have shown that early microspore development is identical in the male sterile mutant “Ougan” mandarin, but there are differences from the tetrad stage until the mature pollen stage (Hu et al., 2007). During abnormal microspore development, microspores with various sizes may develop to the tetrad stage, and they then become less dense and shrunken (Panha et al., 2015). In the present study, we found that the microspores of IAMSLs all exhibited abnormal development from the late uninucleate stage, including irregular appearance, where they eventually became highly vacuolated or degenerate. Thus, limitations on the nutrients and energy supplied to the developing microspores may lead to pollen deformity from the late uninucleate stage. It should be noted that microspores develop adjacent to the tapetum, which provides nutrients for pollen development and materials for pollen wall formation (Xie et al., 2014), and it is a specialized cell layer located between the sporogenous tissue and anther wall (Wang et al., 2015). The appropriate degeneration of tapetal cells guided by PCD supplies sufficient nutrition for the development of microspores, where either delayed or premature PCD in the tapetum results in male sterility (Jung et al., 2005; Ahmadikhah and Karlov, 2006; Li et al., 2006; Zhang et al., 2010; Luo et al., 2013). In previous studies, male sterility was associated with premature tapetal PCD, which has been described in the PET1-CMS sunflower line (Balk and Leaver, 2001), Honglian CMS (HL-CMS) rice line (Li et al., 2004), and TAZ1-silenced plants (Kapoor et al., 2002). Premature PCD was observed in wild abortive CMS (WA-CMS) rice where high levels of ROS occurred in the presence of WA352 (Luo et al., 2013). Interestingly, our results showed that although the two types of IAMSLs have the same nuclear background, the abortive periods and causes were different, where premature PCD occurred in the tapetum in K87B1-706A during the tetrad stage and delayed PCD during the late uninucleate stage in Ju87B1-706A, and the detected fragmentation of nuclear DNA (TUNEL fluorescent signal) appeared earlier than the cytological phenotype in all of the materials, thereby providing some new insights into the molecular mechanism responsible for dysfunctional tapetal PCD.

### ROS and CMS

Mitochondria are important for maintaining the normal functions of cells, which involve the synthesis of nuclear acids and proteins, but also for the initiation of PCD and the response to oxidative stress in aerobic organisms (Balk and Leaver, 2001; Sweetlove et al., 2002; Yi et al., 2016). They are also a major cellular source of ROS due to electron leakage from the electron-transport activity during respiration, and they are among the main targets for attack by ROS under oxidative stress conditions, which may disrupt the normal function of mitochondria due to the accumulation of ROS (Bartoli et al., 2004; Wan et al., 2007; Semwal et al., 2014). Moreover, severe damage to cells can occur when excess ROS are generated, including denaturation of proteins, lipid peroxidation, DNA mutations, and even PCD (Li et al., 2004; Baxter et al., 2014; Vimal et al., 2014). MDA is considered an indicator of lipid peroxidation caused by ROS, and high MDA contents are always related to oxidative stress (Hameed et al., 2013; Noreen et al., 2014). In the present study, we detected rapid and continual increases in the ROS levels in the IAMSLs during the all developmental stages, where the maximum levels occurred in the late uninucleate stage. In addition, we detected continual increases in the MDA contents in the IAMSLs throughout anther development, where the MDA levels were higher than those in the maintainer line in the late uninucleate stage. Previous studies agree with our results, where they showed that excess ROS accumulated in the peak abortion stage and the activities of antioxidant enzymes were decreased significantly, thereby causing an imbalance in the generation and scavenging of ROS (Jian et al., 2015). During the abortion peak (meiosis stage in pollen mother cells), another study found that the ROS contents increased greatly compared with the maintainer line and F1 hybrid, where the levels were much higher than normal (Jiang et al., 2007). In addition, the relationships between ROS production and oxidative stress have been reported, where many abiotic stress factors may cause necrotic lesions and PCD (Petrov et al., 2015). These results demonstrate that ROS overproduction plays an important role during the critical period of microspore abortion and there is probably a close relationship between ROS and pollen development.

### Antioxidant Defense System and CMS

In order to thrive in unfavorable climatic conditions, plants have evolved an antioxidant defense system inside cells, which allows organisms to resist oxidative stress by maintaining the normal production and clearance rate of ROS to reduce their effects on various biological molecules (Moller, 2001). The antioxidant defense system includes enzymatic and non-enzymatic antioxidant components (Ba et al., 2013), where AsA and GSH are the most effective reducing substrates for ROS detoxification (Jiang and Zhang, 2001). AsA is usually regarded as the first line of antioxidant defense because it reacts directly with free radicals and H_2_O_2_. GSH is a substrate for GSH S-transferase and it is the most important non-protein thiol compound, where it is involved in the control of ROS via the AsA-GSH cycle and it yields oxidized GSH (Noctor and Foyer, 1998). In this study, the AsA and GSH contents of 706B were significantly higher than those of the IAMSLs during the entire period of pollen development, especially the late uninucleate stage. Thus, the oxidative stress caused a large number of reducing substances to be oxidized and damaged the AsA-GSH cycle, thereby leading to the accumulation of more ROS. In addition, SOD, POD, CAT APX, and GPX are recognized as important ROS-scavenging enzymes that can eliminate free oxygen radicals in cells (Wan et al., 2007). Nevertheless, direct modification of the expression levels of enzymes in chloroplasts is considered to a key factor involved with the protection of plants against various oxidative stresses (Badawi et al., 2004). Therefore, the antioxidant system plays a major role in maintaining the redox state of plant cells. Naturally, SOD is a free radical scavenger in plant cells, where it can convert harmful O_2_^-^ into H_2_O_2_ and O_2_. H_2_O_2_ is also a harmful ROS in cells, but CAT and POD can immediately transform H_2_O_2_ into H_2_O. Thus, these three enzymes form a complete antioxidant chain and they work together to resist oxidative stress (Arora et al., 2002). We demonstrated that the activities of antioxidant enzymes were affected by abnormal oxygen metabolism. In particular, throughout the anther development process, the SOD, CAT, and POD activity levels were higher in the IAMSLs than those in 706B, where there was a highly significant difference between the levels in the IAMSLs and 706B in the late uninucleate stage, as found in a previous study (Song et al., 2008; Liu et al., 2011). In addition, the CAT activity levels generally tended to decrease in the IAMSLs, whereas the opposite occurred in 706B during the late uninucleate stage to the trinucleate stage. It is possible that the increased generation of O_2_^-^ and H_2_O_2_ triggered the activation of the antioxidant system, thereby enhancing the SOD, CAT, and POD activities to protect the cells. Studies of CMS plants have shown that compared with the control, plants with the spikelets removed and those without spikelets had higher antioxidant enzyme activities, better antioxidant defenses, and less oxidative damage (Srivalli and Khanna-Chopra, 2004, 2009). However, we found that compared with 706B, the APX and GPX contents were lower in the IAMSLs in the binucleate stage and late uninucleate stage, respectively. Therefore, during the later period for pollen abortion, in addition to the increases in the O_2_^-^, H_2_O_2_, and MDA contents, the APX and GPX activities as well as the AsA and GSH contents were lower in the IAMSLs. Similar results were obtained by Shi et al. (2001). Therefore, according to our analysis, we suggest that active oxygen metabolism was imbalanced and the peroxidation of membrane lipids was enhanced due to the disordered antioxidant defense system in the IAMSLs, where the irregular shapes of the microspores could have been related to the abnormal active oxygen metabolism.

The excessive accumulation of ROS can also affect the expression of various biological molecules via oxidative damage. Many studies have shown that mitochondrial ROS can act as signals that affect nuclear gene expression via mitochondria-to-nucleus signaling, which is referred to as mitochondrial retrograde regulation (Dancy et al., 2014; Scheibye-Knudsen et al., 2015; West et al., 2015). In the present study, in order to determine the expression levels of the genes encoding antioxidant enzymes, we optimized the PCR reaction conditions and conducted qRT-PCR. The expression levels of the SOD and APX genes were similar to the enzyme activity levels, but there were difference between the CAT gene expression levels and CAT enzyme activities, possibly because multiple CAT genes were present. In this study, we obtained new insights into PCD in plants by analyzing the mechanism responsible for male sterility using approaches based on cell biology, physiology, and molecular biology, and our results may also facilitate the development of new IAMSLs by eliminating the influence of the nucleus. These results provide the necessary theoretical basis for further research into how the ROS generation system interacts with the tapetal transcriptional network during anther abortion.

## Conclusion

In this study, we explored the abortion mechanism in two types of economically important IAMSLs conferred by cytoplasm from *Ae. kotschyi* and *Ae. juvenalis*, where we found that excessive ROS and the abnormal transcript levels of *SOD, CAT*, and *APX* genes may disrupt the antioxidative balance, thereby initiating aberrant tapetal PCD, with premature PCD in the tapetum of K87B1-706A during the tetrad stage and delayed PCD in Ju87B1-706A during the late uninucleate stage, which ultimately results in pollen abortion. These relationships between the mechanism of PCD and ROS metabolism provide new insights into the mechanisms responsible for abortive pollen in wheat, as well as the necessary theoretical basis for generating commercially valuable male sterile plants.

## Author Contributions

XSo, ZL, LZ, and XSh conceived and designed the study. XSo critically revised the manuscript. All of the authors read and approved the manuscript.

## Conflict of Interest Statement

The authors declare that the research was conducted in the absence of any commercial or financial relationships that could be construed as a potential conflict of interest.
