# Supplementary material for: Oxidative Stress and Aberrant Programmed Cell Death Are Associated With Pollen Abortion in Isonuclear Alloplasmic Male-Sterile Wheat
Source: Front Plant Sci. 2018 May 4;9:595. doi: 10.3389/fpls.2018.00595 (PMC5945952; doi:10.3389/fpls.2018.00595)
Supplement: Supplementary file 1 [file Table_1.docx]

**Supplemental Information**

**Table S1** Primers used for quantitative real-time RT-PCR analysis of antioxidant genes related to CMS

| Primer | Sequence (5′-3′) | T_m_/°C |
| --- | --- | --- |
| SOD-f  SOD-r | AGAACCTCAAGCCTATCAGC  GACAAATCACGCAAGCACT | 60 |
| CAT-f  CAT-r | TGCCTGTGTTTTTTATCCGA  ACCGTCCATGTGCCTGTAGT | 62 |
| APX-f  APX-r | GTTCATCCCTGGAAGACG  CAGAGGGTCACGAGTCCA | 64 |
| Actin-f  Actin-r | CTCCCTCACAACAACCGC  TACCAGGAACTTCCATACCAAC | 62 |
