# Supplementary material for: Oxidative Stress and Aberrant Programmed Cell Death Are Associated With Pollen Abortion in Isonuclear Alloplasmic Male-Sterile Wheat
Source: Front Plant Sci. 2018 May 4;9:595. doi: 10.3389/fpls.2018.00595 (PMC5945952; doi:10.3389/fpls.2018.00595)

**Supplemental Information**

**Figure S1** Proportions of different abortion types in IAMSLs.

**Figure S2** Numbers of Ubisch bodies and statistical differences in the IAMSLs and fertile wheat.

**Figure S3** Observations of the anther tapetum in K87B1-706A (A–E), Ju87B1-706A (F–J), and 706B (K-O) during different developmental stages. Tds, tetrad stage (A, F, and K); Ens, early uninucleate stage (B, G, and L); Lns, late uninucleate stage (C, H, and M); Bns, binucleate stage (D, I, and N); and Tns, trinucleate stage (E, J, and O). E, epidermis; En, endothecium; ML, middle layer; T, tapetum; Tds: tetrads; Msp: microspores. Scale bars = 50 μm (A–O).

**
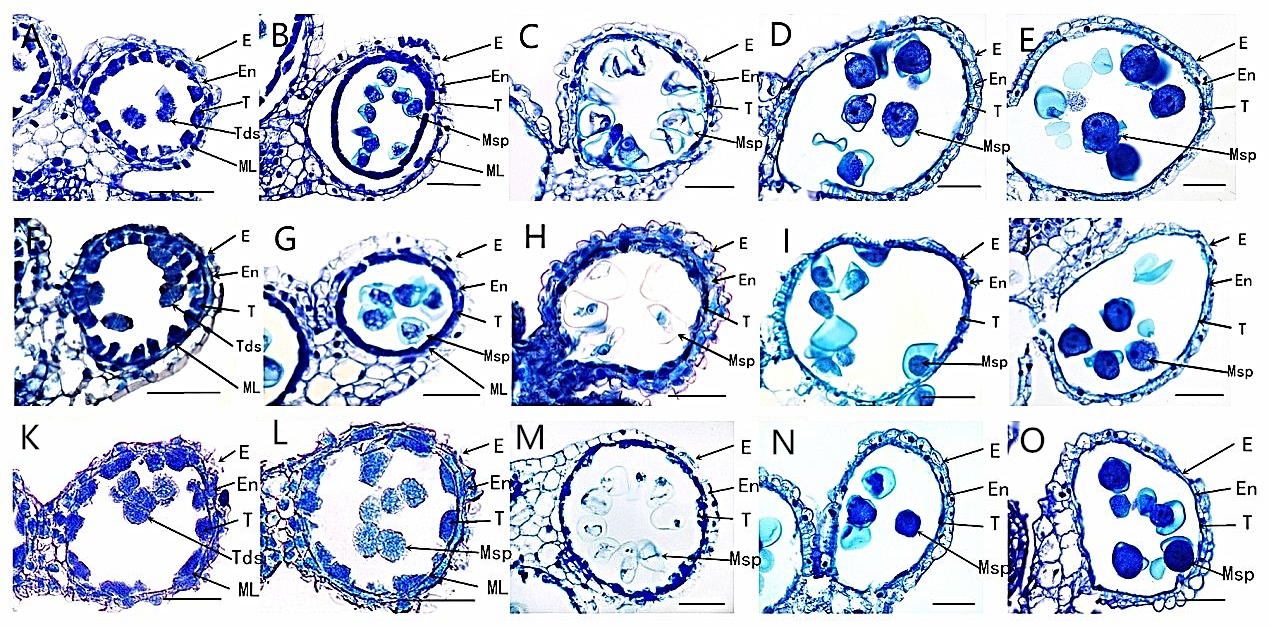
**

**Figure S4** Sectional areas of the tapetum cells in K87B1-706A, Ju87B1-706A, and 706B. Tds, tetrad stage; Ens, early uninucleate stage; Lns, late uninucleate stage.


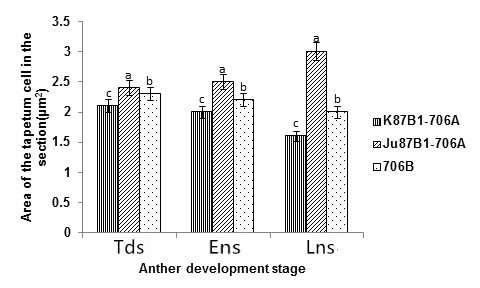


**Figure S5** Detection of DNA laddering in the anther tapetum from K87B1-706A, Ju87B1-706A, and 706B during different developmental stages. M, Marker D2000; Tds, tetrad stage; Ens, early uninucleate stage; Lns, late uninucleate stage; Bns, binucleate stage; and Tns, trinucleate stage.

K87B1-706A

706B

Ju87B1-706A


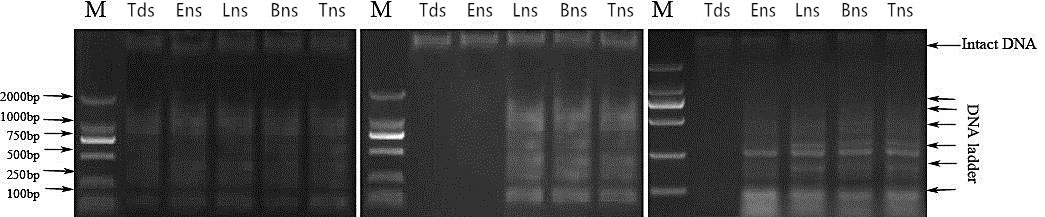


**Figure S6** Analysis of the correlations between the *SOD* enzyme gene expression levels and SOD enzyme activities in the IAMSLs and fertile wheat during different developmental stages.


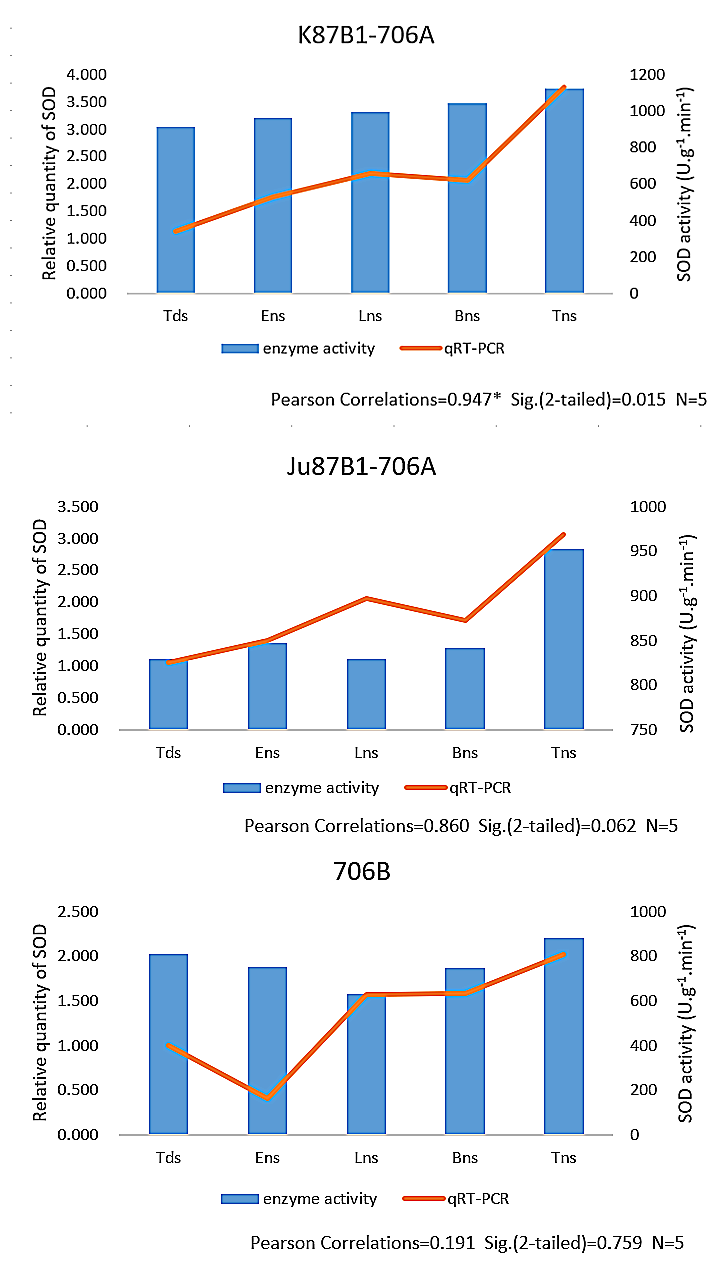


**Figure S7** Analysis of the correlations between the *CAT* enzyme gene expression levels and CAT enzyme activities in the IAMSLs and fertile wheat during different developmental stages.


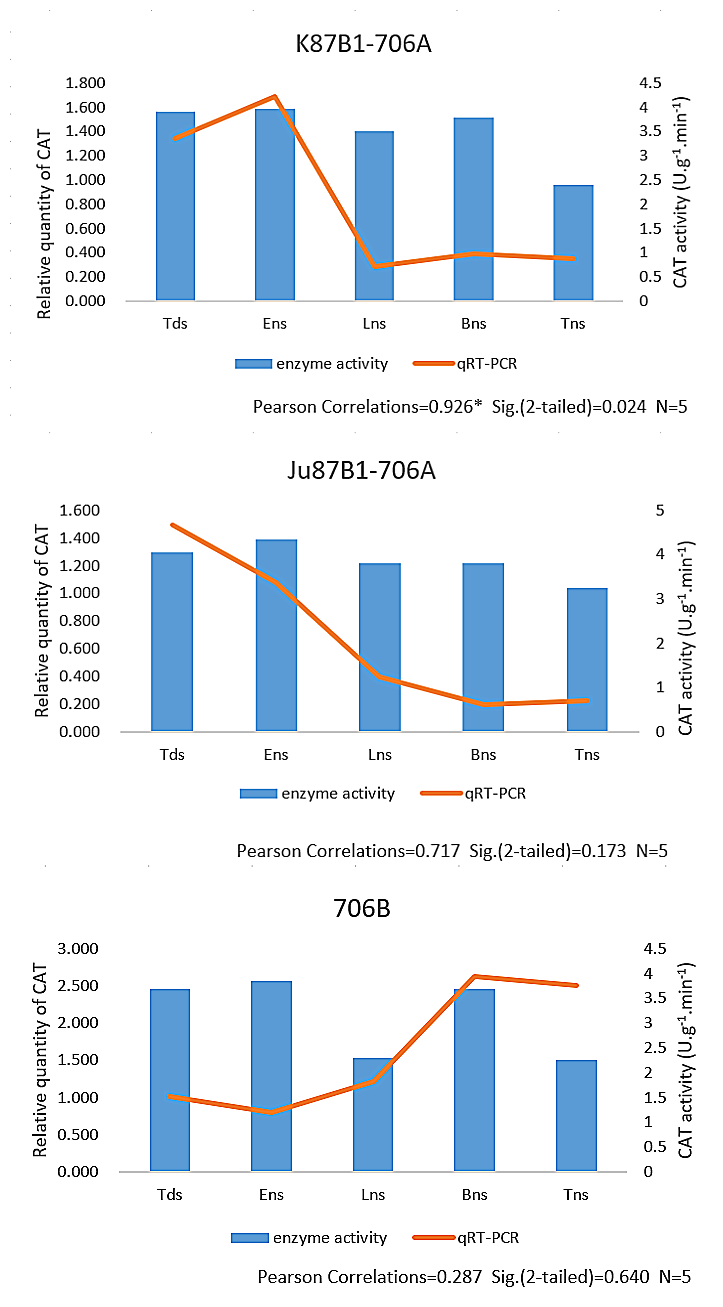


**Figure S8** Analysis of the correlations between the *APX* enzyme gene expression levels and APX enzyme activities in the IAMSLs and fertile wheat during different developmental stages.


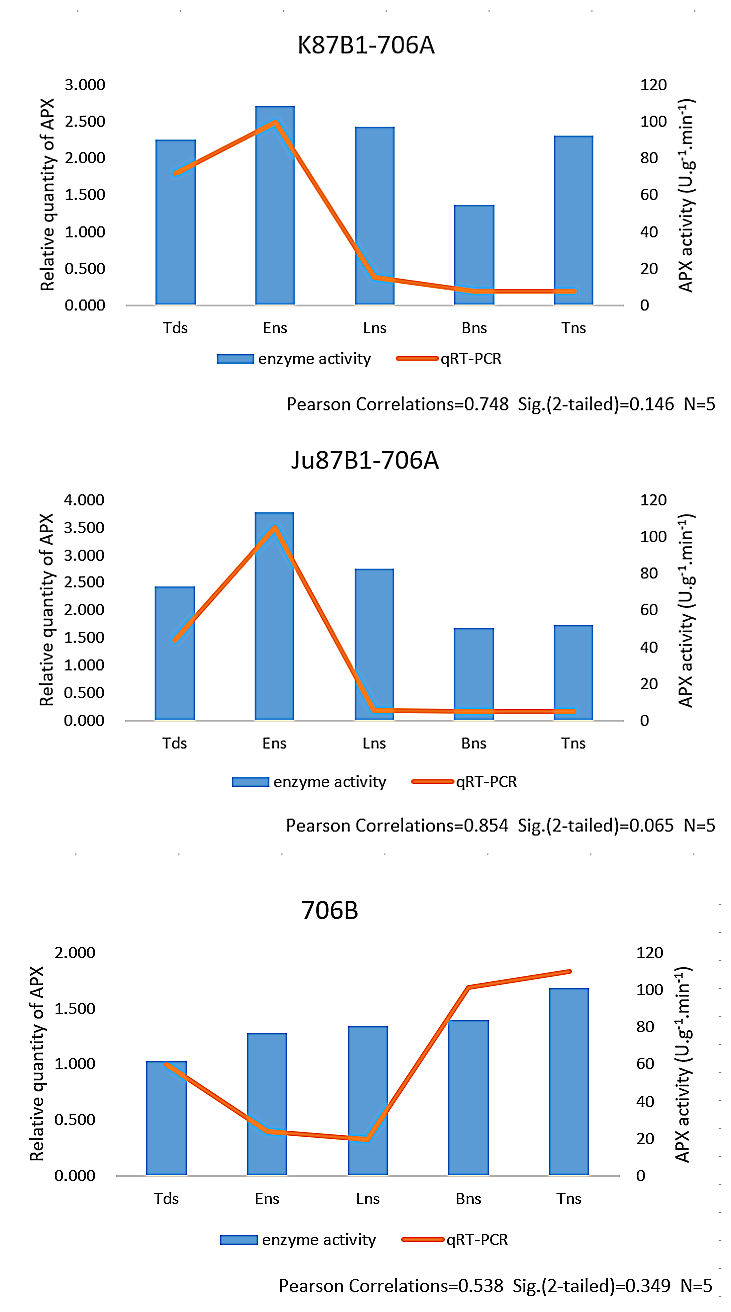

Supplement: Supplementary file 2 [file Data_Sheet_1.docx]
